# Defining genetic determinants of the Metabolic Syndrome in the Framingham Heart Study using association and structural equation modeling methods

**DOI:** 10.1186/1753-6561-3-s7-s50

**Published:** 2009-12-15

**Authors:** Nora L Nock, Xuefeng Wang, Cheryl L Thompson, Yeunjoo Song, Dan Baechle, Paola Raska, Catherine M Stein, Courtney Gray-McGuire

**Affiliations:** 1 Department of Epidemiology and Biostatistics, Case Western Reserve University, 2103 Cornell Road, Cleveland, Ohio 44106 USA; 2 Center for Transdisciplinary Research on Energetics and Cancer, 10900 Euclid Avenue, Case Western Reserve University, Cleveland, Ohio 44106 USA; 3 Department of Family Medicine, Case Western Reserve University, 11100 Euclid Avenue, Cleveland, Ohio 44106 USA

## Abstract

The Metabolic Syndrome (MetSyn), which is a clustering of traits including insulin resistance, obesity, hypertension and dyslipidemia, is estimated to have a substantial genetic component, yet few specific genetic targets have been identified. Factor analysis, a sub-type of structural equation modeling (SEM), has been used to model the complex relationships in MetSyn. Therefore, we aimed to define the genetic determinants of MetSyn in the Framingham Heart Study (Offspring Cohort, Exam 7) using the Affymetrix 50 k Human Gene Panel and three different approaches: 1) an association-based "one-SNP-at-a-time" analysis with MetSyn as a binary trait using the World Health Organization criteria; 2) an association-based "one-SNP-at-a-time" analysis with MetSyn as a continuous trait using second-order factor scores derived from four first-order factors; and, 3) a multivariate SEM analysis with MetSyn as a continuous, second-order factor modeled with multiple putative genes, which were represented by latent constructs defined using multiple SNPs in each gene. Results were similar between approaches in that *CSMD1 *SNPs were associated with MetSyn in Approaches 1 and 2; however, the effects of *CSMD1 *diminished in Approach 3 when modeled simultaneously with six other genes, most notably *CETP *and *STARD13*, which were strongly associated with the Lipids and MetSyn factors, respectively. We conclude that modeling multiple genes as latent constructs on first-order trait factors, most proximal to the gene's function with limited paths directly from genes to the second-order MetSyn factor, using SEM is the most viable approach toward understanding overall gene variation effects in the presence of multiple putative SNPs.

## Background

The Metabolic Syndrome (MetSyn) is a clustering of metabolic disturbances that increases the risk of type 2 diabetes and cardiovascular disease [1], and may contribute to the pathogenesis of other complex diseases, including cancer [2]. MetSyn is estimated to affect over 47 million adult Americans [3,4] and is becoming increasingly more prevalent worldwide [5,6]. Although MetSyn has been shown to increase with age, recent studies have shown a rise in this disease among younger people in the U.S., particularly in women 20 to 39 years of age [1]. Interestingly, this rise mirrors the increasing rates of obesity in women of these ages.

Although it is well established that MetSyn involves the co-occurrence of multiple metabolic traits, there are differences in the formal definitions set forth by the World Health Organization (WHO), the National Cholesterol Education Program Third Adult Treatment Panel (NCEP-ATP III), the American Heart Association/National Heart, Lung and Blood Institute (AHA/NHLBI) and the International Diabetes Federation (IDF), predominantly in defining the most relevant elements and their biological cut-points, which has contributed to confusion in the literature [7]. Nevertheless, all of these definitions include criteria on four common traits: 1) insulin resistance, 2) obesity, 3) hypertension, and 4) dyslipidemia. Factor analysis, a statistical method under the umbrella of structural equation modeling (SEM), has been used, albeit sparingly, to help define the critical elements and structure of the syndrome. Studies conducted in adults using 8 to 10 metabolic measures (fasting insulin, fasting glucose, post-challenge insulin, post-challenge glucose, body mass index (BMI), waist circumference or waist-to-hip ratio (WHR), high density lipoprotein-cholesterol (HDL), triglycerides (TG), systolic blood pressure (SBP), diastolic blood pressure (DBP)) have shown that the MetSyn is best described, statistically, as a unifying, second-order factor defined by four first-order factors (Insulin Resistance, Obesity, Hypertension, Lipids) [8-10].

MetSyn is hypothesized to have fairly large genetic component with heritability estimates ranging from 6.3% to 50% [11], yet few potential genetic targets have been identified. Thus, we aimed to define the genetic determinants of MetSyn in the Framingham Heart Study (Offspring Cohort, Exam 7) using the Affymetrix 50 k Human Gene Panel data and three different approaches: 1) an association-based "one-single-nucleotide polymorphism (SNP)-at-a-time" analysis with MetSyn defined as a binary trait using the WHO criteria [7]; 2) an association-based "one-SNP-at-a-time" analysis with MetSyn defined as a continuous trait using second-order factor scores derived from insulin resistance, obesity, hypertension, and dyslipidemia factors; and, 3) a multivariate SEM analysis with MetSyn defined as a second-order continuous factor trait modeled simultaneously with putative genes (identified in Approaches 1 and 2), which we represented as latent constructs defined by multiple SNPs within each gene.

## Methods

### Data cleaning and preparation: phenotype and genotype variables

First, we examined the distribution of metabolic variables (TG, HDL, SBP, DBP, fasting glucose, BMI) in the Offspring Cohort, Exam 7 using SAS v9.1 (SAS Institute Inc., Cary, NC). Variables not following a normal distribution, as determined by visual inspection of histograms and quantile-quantile plots and formal Shapiro-Wilk's and Kolmogorov-Smirnov tests, were natural log transformed (TG, HDL, fasting glucose). To adjust for potential bias by antihypertensive treatment and more closely reflect pretreatment BP values, we added 10 mm Hg to SBP and 5 mm Hg to DBP, following Cui et al. [12], in subjects who reported taking blood pressure medications. We used the WHO criteria [7] to define MetSyn; however, waist circumference and microalbuminuria measures were not available, and we applied the most recent IDF fasting glucose cut-point value of ≥100 mg/dL [7]. Mendelian inconsistencies were identified in the Affymetrix 50 k Human Gene Panel data using MARKERINFO (S.A.G.E. v5.4.1). If an inconsistency was found, genotypes of all individuals in that family were set to missing. Of the 2760 subjects in the Offspring Cohort (Approach 1), 2544 had complete data on all metabolic measures (Approach 2) and 1512 had complete data on all metabolic measures and putative genotypes (Approach 3).

### Statistical methods for association-based analyses

#### Approach 1 and 2

We evaluated the potential association between each SNP on the 50 k panel and each phenotype using the following model [13] in ASSOC (S.A.G.E. v5.4.1):

where for any individual *i*, with trait *y*_*i*_, *c*_*ji *_is any one of *n *individual specific covariates, *η*_*i *_is a random effect comprising, in our analyses, the sibling and individual specific errors, *z*_*i *_is a genotype indicator for allele A at a diallelic locus with alleles A and B:

where *h *is the generalized modulus power transformation [14], which estimates the regression coefficients, *γ*_*j *_and *δ*, as median unbiased on the original scale of measurement. Analyses were adjusted for age, sex and age × sex. *p*-Values were calculated using likelihood-ratio and Wald tests and compared to ensure consistency; however, we report only the Wald *p*-values since results were similar in all cases. In Approach 1 and 2, a gene was considered statistically significant if it had ≥2 SNPs associated with an individual metabolic variable or MetSyn at *p *< 0.001. Significant genes were then utilized in Approach 3.

### Statistical methods for factor analysis (FA) and SEM

#### Approach 2 and 3

We used previous reports to devise our second-order MetSyn factor model [9,10]; however, because fasting insulin and waist circumference were not available, first-order factors, Insulin Resistance and Obesity were defined using only fasting glucose and BMI, respectively. Similar to previous models [9,10], the BP and Lipids first-order factors were defined using SBP and DBP and TG and HDL measures, respectively. We performed confirmatory factor analysis using a robust maximum likelihood estimator (MLR), which provides test statistics and standard errors robust to non-independence of observations and non-normality (Mplus v5.1; TYPE = COMPLEX), to formally test our second-order MetSyn model and to generate corresponding factor scores. In Approach 2, we examined potential associations between each SNP on the 50 k panel and the factor scores with ASSOC (S.A.G.E. v5.4.1). In Approach 3, we extended the latent gene construct SEM method of Nock et al. [15] using the robust maximum likelihood estimator (MLR; Mplus v5.1) to simultaneously model MetSyn as a second-order factor together with multiple putative genes identified in Approaches 1 and 2. Similar to Nock et al. [15], we used eigenvalues, scree plots, factor patterns, Cronbach's alpha and linkage disequilibrium (LD) plots (Haploview v4.1) to help select the most informative SNPs in devising the latent gene constructs. For putative genes identified in Approaches 1 and 2, we utilized all available SNPs on the 50 k panel, including those SNPs found to be statistically significant in Approaches 1 and 2, unless they provided redundant information and created a linear dependency. To assess the overall model goodness-of-fit to the data, the *χ*^2 ^test, comparative fit index (CFI), root mean square error of approximation (RMSEA) and standardized root mean square residual (SRMR) were evaluated [16]. The *χ*^2 ^test, which evaluates whether the covariance matrix is equal to the model-implied covariance matrix predicted by the parameters, is very sensitive to sample size and complexity. Thus, other fit indices such as the CFI, RMSEA, and SRMR have been proposed as alternative descriptive measures for evaluating model fit [16]. Values for the CFI, which is relatively insensitive to sample size and model complexity, of ≥0.90 and ≥0.95 indicate acceptable and good fit, respectively [17]. Values for the RMSEA (an index less sensitive to sample size and favoring more parsimonious models) values of ≤0.06 represent good fit while values >0.10 represent unacceptable fit [17]. A SRMR ≤0.08 and <0.10 represent good and acceptable fit, respectively [16,17]. All *p*-values are from two-sided tests and statistical significance set at *p *≤ 0.05 in Approach 3.

## Results

In Approach 1, we evaluated the potential associations between each SNP on the Affymetrix 50 k Human Gene Panel and MetSyn as a binary trait defined using the modified WHO criteria (see Methods) and between each SNP and each individual metabolic measure. We found that several genes had two or more SNPs with significant (*p *< 0.001) associations, the majority of which are listed in Table 1. Of these genes, the most significant associations were observed with *CETP *SNPs rs11508026 (*p *= 7.60 × 10^-7^) and rs3764261 (*p *= 1.34 × 10^-6^). Two of the same *KIAA0329 *SNPs (rs12434098, rs1190547) were associated with TG and HDL but not with MetSyn. Multiple *CSMD1 *SNPs were associated with BMI and MetSyn, while *WDR64 *SNPs were associated with SBP and MetSyn.

**Table 1:** Genes with ≥ 2 SNPs associated with individual metabolic measures and MetSyn^a ^at *p *< 0.001

Trait and Chr	Gene symbol	Gene ID	rs number	Base pair/AA change	MAF	β (S.E.)	*p*-Value
Fasting glucose							
Chr 13	*STARD13*	90627	515192	Outside G/T	0.400	0.034 (0.009)	0.000266
	*STARD13*	90627	2858808	Intron C/T	0.364	0.032 (0.009)	0.000808
BMI							
Chr 4	*KCTD8*	386617	13143747	Thr/Thr	0.092	-1.612 (0.419)	0.000118
	*KCTD8*	386617	17599556	Intron A/C	0.142	-0.978 (0.276)	0.000371
	*KCTD8*	386617	2347926	Intron A/C	0.242	-1.066 (0.299)	0.000400
Chr 8	*CSMD1*	64478	1997137	Intron G/T	0.167	0.944 (0.281)	0.000284
	*CSMD1*	64478	2930355	Intron A/G	0.183	1.241 (0.342)	0.000778
Chr 13	*-*	729646	311865	Outside C/T	0.267	1.293 (0.265)	0.000001
	*-*	729646	1006255	Outside C/T	0.417	-0.933 (0.240)	0.000102
TG							
Chr 14	*KIAA0329*	9895	1210074	Intron A/G	0.246	0.093 (0.027)	0.000462
	*KIAA0329*	9895	12434098	Intron C/T	0.475	0.101 (0.029)	0.000682
	*KIAA0329*	9895	1190547	Intron C/G	0.242	0.091 (0.028)	0.000966
HDL							
Chr 14	*KIAA0329*	9895	12434098	Intron C/T	0.475	-0.050 (0.014)	0.000322
	*KIAA0329*	9895	1190547	Intron C/G	0.242	-0.049 (0.014)	0.000637
Chr 16	*CETP*	1071	11508026	Intron C/T	0.492	-0.075 (0.016)	7.60 × 10^-7^
	*CETP*	1071	3764261	Outside G/T	0.367	-0.069 (0.014)	1.34 × 10^-6^
SBP							
Chr 1	*WDR64*	128025	12074374	Trp/Arg	0.208	-4.271 (1.207)	0.000246
	*WDR64*	128025	12095445	Gln/Arg	-	-4.390 (1.197)	0.000402
Chr 13	*MYO16*	23026	4772992	Intron A/G	0.383	-2.895 (0.876)	0.000158
	*MYO16*	23026	6492144	Intron C/G	0.392	-2.964 (0.785)	0.000777
	*MYO16*	23026	9514889	Intron A/G	0.192	-2.673 (0.795)	0.000953
MetSyn^a^							
Chr 1	*WDR64*	128025	12074374	Trp/Arg	0.208	-0.060 (0.018)	0.000721
	*WDR64*	128025	12095445	Gln/Arg	-	-0.061 (0.018)	0.000961
Chr 8	*CSMD1*^b^	64478	7013078	Intron A/C	0.040	-0.135 (0.039)	0.000582
	*CSMD1*	64478	12549291	Intron G/T	0.250	0.045 (0.013)	0.000808

^a^MetSyn defined as binary trait using a modified WHO definition (see text).

^b^rs1997137 and rs2930355 in *CSMD1 *were associated with MetSyn at *p *= 0.007 and *p *= 0.003, respectively.

**Figure 1:**
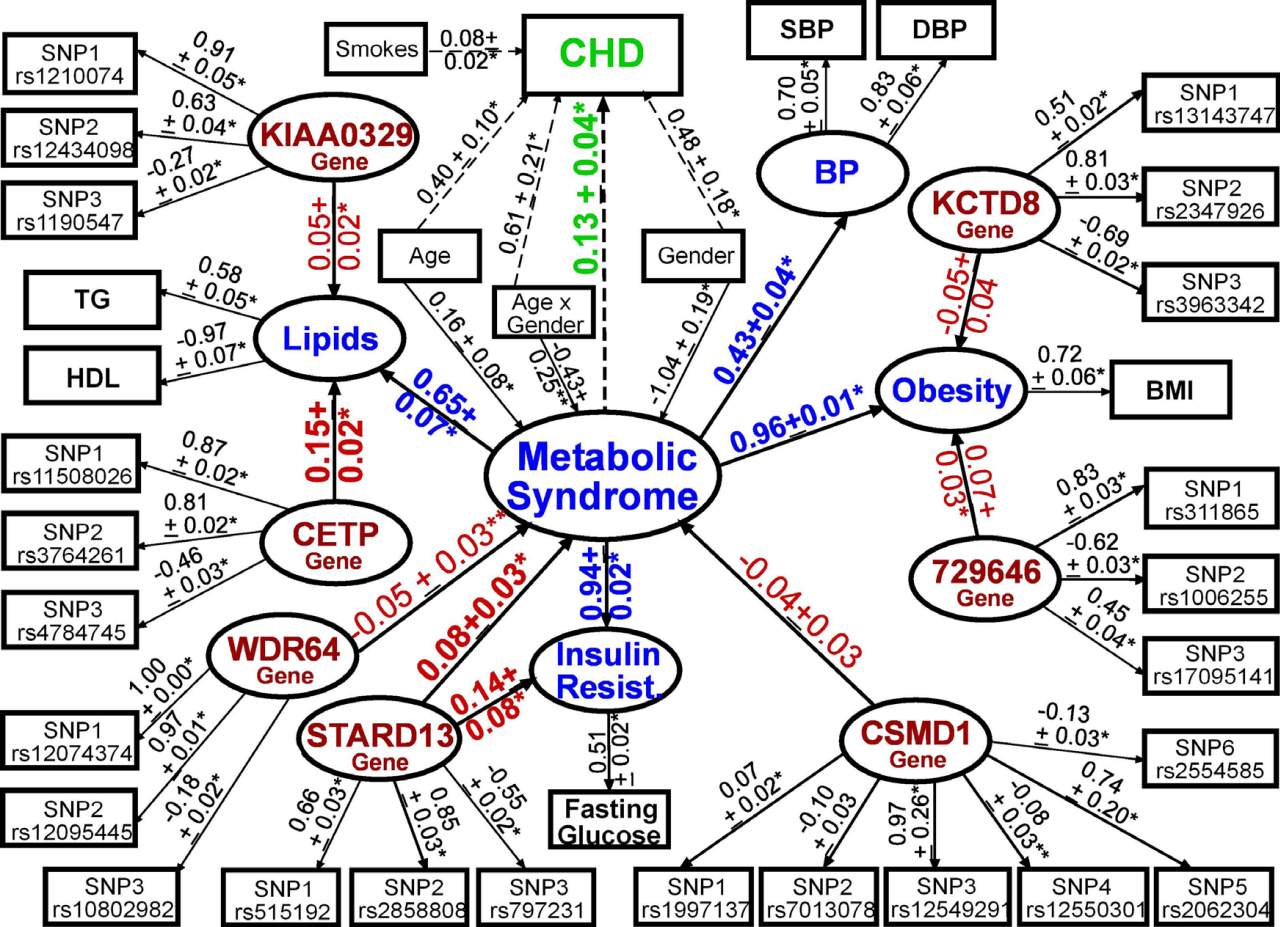
**Model of the MetSyn and genes as latent constructs using SEM**. Model resulted in good overall model fit (*χ*^2 ^= 1336.00, df = 457, *p *< 0.05; CFI = 0.02; RMSEA = 0.03; SRMR = 0.03). Standardized loadings and corresponding standard errors are depicted above arrows. Blue, MetSyn traits; Red, Genes; Green, coronary heart disease; *p ≤ 0.05; **p ≤ 0.10. Residuals not shown for clarity.

In Approach 2, we modeled MetSyn as a continuous, second-order factor defined by four first-order factors (Obesity, Insulin Resistance, BP, Lipids) and observed a good fitting model by several fit indices (*χ*^2 ^= 59.48, df = 7, *p *< 0.05; CFI = 0.97; RMSEA = 0.05; SRMR = 0.02). We found Insulin Resistance was the most important factor (*β*_std _= 0.99 ± standard error of *β*_std _= 0.02; *p *< 0.001) followed by Obesity (0.96 ± 0.03; *p *< 0.001), Lipids (0.69 ± 0.05; *p *< 0.001), and BP (0.50 ± 0.04; *p *< 0.001). Using this model, we examined potential associations between each SNP on the 50 k panel and the second- and first-order factor scores (Table 2). *KIAA0329 *SNPs were found to be associated with Lipids factor scores (Table 2), which is consistent with the results observed in Approach 1 in that *KIAA0329 *SNPs were also associated with TG and HDL measures. However, *CSMD1 *was the only gene with ≥2 SNPs found to be associated with MetSyn factor scores (*p *< 0.001). In Approach 3, we extended the latent gene construct SEM method of Nock et al. [15] to model multiple putative genes identified in Approaches 1 and 2 simultaneously with MetSyn as a second-order factor modeling MetSyn as second-order factor with 24 SNPs in seven genes improved model fit by several indices (*χ*^2 ^= 523.52, df = 375, *p *< 0.05; CFI = 0.99; RMSEA = 0.01; SRMR = 0.02) and increased the *R*^2 ^of MetSyn from 0.23 to 0.43, compared to the same model without genes. To further illustrate the utility of SEM, we also added a path between MetSyn and coronary heart disease (CHD) in models with and without genes. As shown in Figure 1, the strongest associations in terms of effect size and statistical significance were found between *CETP *and Lipids (*β*_std _= 0.15 ± 0.02; *p *= 1.04 × 10^-8^), *STARD13 *and Insulin Resistance (*β*_std _= 0.14 ± 0.08; *p *= 0.05), and *STARD13 *and MetSyn (*β*_std _= 0.08 ± 0.03; *p *= 0.007); however, the *CSMD1 *latent construct was not associated with MetSyn even when using different combinations of SNPs to devise the construct (data not shown). The *MYO16 *latent gene construct was not associated with either BP or MetSyn factors; and, for parsimony reasons, was dropped from the final model shown in Figure 1. The association between MetSyn and CHD was similar, but slightly attenuated in the model with (*β*_std _= 0.13 ± 0.04; *p *= 0.002) versus without (*β*_std _= 0.14 ± 0.03; *p *< 0.001) the seven genes.

**Table 2:** Genes with ≥ 2 SNPs associated with MetSyn factor scores^a ^at *p *< 0.001

Trait and Chr	Gene symbol	Gene ID	rs number	Base pair change	MAF	β (S.E.)	*p*-value
Insulin resistance (first-order factor)							
Chr 8	*CSMD1*	64478	7013078	Intron A/C	0.040	-4.724 (1.392)	0.000594
	*CSMD1*	64478	1997137	Intron G/T	0.167	2.572 (0.755)	0.000623
Obesity (first-order factor)							
Chr 8	*CSMD1*	64478	1997137	Intron G/T	0.167	0.674 (0.194)	0.000469
	*CSMD1*	64478	7013078	Intron A/C	0.040	-1.222 (0.360)	0.000510
Lipids (first-order factor)^b^							
Chr 14	*KIAA0329*	9895	1210074	Intron A/G	0.246	0.230 (0.063)	0.000285
	*KIAA0329*	9895	12434098	Intron C/T	0.475	0.211 (0.060)	0.000420
	*KIAA0329*	9895	1190547	Intron C/G	0.242	0.304 (0.129)	0.000585
MetSyn (second-order factor)^a^							
Chr 8	*CSMD1*	64478	1997137	Intron G/T	0.167	0.189 (0.056)	0.000583
	*CSMD1*	64478	7013078	Intron A/C	0.040	-0.428 (0.103)	0.000632

^a^Scores from a second-order, MetSyn factor model (factors shown in blue text only in Figure 1 depict a similar model).

^b^No genes with two or more SNPs were found to be associated with the BP first-order factor at *p *< 0.001.

## Discussion

Results between approaches were similar in that *CSMD1 *SNPs were found to be associated with MetSyn when using both the modified WHO definition (Approach 1) and the factor scores (Approach 2). However, when evaluating associations between each SNP and each individual metabolic measure, the factor scores (Approach 2) produced fewer putative genes with ≥2 SNPs using a *p *< 0.001. Because we were most interested in defining putative genes and not individual SNPs, we retained genes with ≥2 SNPs at *p *< 0.001 versus correcting for multiple tests using a standard Bonferroni correction approach. If we had applied a correction factor for 2,000 tests (*p *≤ 2.5 × 10^-5^), which is the approximate number of genes on the 50 k panel, only *CETP *(mean SNP *p *= 1.05 × 10^-6^) would have qualified for use in Approach 3. Interestingly, the *CETP *latent gene construct (Approach 3) had the strongest association of all of the gene constructs in terms of effect size (*β*_std _= 0.15) and significance (*p *= 1.04 × 10^-8^) in the 7-gene, 24-SNP model (Figure 1).

Although *CSMD1 *SNPs were associated with MetSyn in Approaches 1 and 2, the *CSMD1 *latent gene construct (Approach 3) was not associated with Metsyn when modeled in the presence of SNPs in six other genes, even when devising the construct with different combinations of SNPs, which emphasizes an important advantage of Approach 3 in that it can better control for the effects of multiple putative SNPs (and genes) in the same model. Although sample sizes differed between approaches, the consistent findings we observed across all three approaches for *CETP *(HDL (Approach 1), Lipids (Approach 2)) and *STARD13 *(fasting glucose (Approach 1), Insulin Resistance (Approach 3)) make attributing the *CSMD1 *discrepancies to sample size differences less compelling. Moreover, SNPs in genes previously shown to be associated with MetSyn using the WHO criteria, including *LDLR*, *PPARG*, and *ACE *[18], were not found to be significant at *p *≤ 0.05 in our study. The lack of replication may be due to modifications we had to make to the WHO definition to accommodate available data and, perhaps, genetic heterogeneity of this complex phenotype.

## Conclusion

The multivariate framework of SEM is inherently better suited for modeling the hierarchical, complex relations involved in MetSyn; and, the latent gene construct SEM approach appears particularly useful for disentangling the influence of individual genes on MetSyn in the presence of multiple putative SNPs.

## List of abbreviations used

BMI: Body mass index; CHD: Coronary heart disease; CFI: Comparative fit index; DBP: Diastolic blood pressure; FA: Factor analysis; HDL: High density lipoprotein-cholesterol; IDF: International Diabetes Federation; LD: Linkage disequilibrium; MetSyn: Metabolic Syndrome; RMSEA: Root mean square error of approximation; SBP: Systolic blood pressure; SEM: Structural equation modeling; SNP: Single-nucleotide polymorphism; SRMR: Standardized root mean square residual; WHO: World Health Organization.

## Competing interests

The authors declare that they have no competing interests.

## Authors' contributions

NLN conceived the study, conducted the statistical analyses, and prepared the manuscript. XW, YS, DB, CT, and PR cleaned the data and helped conduct analyses for Approach 1. CG-M and CS helped with study design and coordination and drafting the manuscript.
